# Transactions of the American Medical Association

**Published:** 1859-12

**Authors:** 


**Transactions of the American Medical Association.---Vol. XII, 1859.**

We have received the twelfth volume of the transactions of the American Medical Association, containing 722 pages, over 400 of which are taken up with what we understand to be a part of the transactions of the Medical Association of Georgia, at their meeting of April, 1859, in the City of Atlanta. The paper to which we allude, bears the title of "Observations on some of the Physical, Chemical, Physiological, and Pathological Phenomena of Malarial Fever, by Joseph Jones, A. M., M. D., Professor of Chemistry in the Medical College of Georgia, at Augusta. If this fact had been acknowledged, it would doubtless have been considered as a compliment by our State Association, (which, not issuing a regular volume of transactions, had authorized their publication in the various Medical Journals of the State,) but under the circumstances, we can not regard the proceeding in any other light, than one of very questionable taste and propriety, and can not forbear to express our surprise.

The volume altogether, can not, we think, be regarded, as in any high degree, creditable to such an able body, as the American Medical Association is acknowledged to be.

